# Tracking the Ghosts of the Himalayas: Snow Leopard Conservation Insights From Satellite Collar Data

**DOI:** 10.1002/ece3.70802

**Published:** 2025-01-06

**Authors:** Pratistha Shrestha, Dayaram Pandey, Pemba Sherpa, Prakash Shah, Dipesh Kumar Sharma

**Affiliations:** ^1^ Central Department of Zoology, Institute of Science and Technology Tribhuvan University Kathmandu Bagmati Nepal; ^2^ Department of National Parks and Wildlife Conservation (DNPWC) Ministry of Forests and Environment, Government of Nepal Kathmandu Bagmati Nepal; ^3^ Département de Biologie, Chimie et Géographie Université du Québec à Rimouski Rimouski Quebec Canada; ^4^ Forest Research and Training Centre (FRTC) Ministry of Forests and Environment, Government of Nepal Kathmandu Bagmati Nepal

**Keywords:** coexistence, hidden Markov model, spatiotemporal dynamics, telemetry, transboundary cooperation

## Abstract

This study presents the first movement analysis of snow leopards ( *Panthera uncia* ) using satellite telemetry data, focusing on the northeastern Himalayas of Nepal. By examining GPS‐based satellite collar data between 2013 and 2017 from five collared snow leopards (effectively three individuals), the research uncovered distinct movement patterns, activity budgeting and home range utilisation from one adult male and two sub adult females. Hidden Markov models (HMMs) revealed three behavioural states based on the movement patterns—slow (indicative of resting), moderate and fast (associated with travelling) and demonstrated that the time of day influenced their behavioural state. While adult males exhibited behaviour focused on moderately active states, juvenile females presented behaviour focused on highly active states. Home ranges, estimated over a 5–21 month tracking period, were larger than those observed in previously studied snow leopards and included crossings of international boundaries from Nepal into China and India. These relatively large home ranges may be attributed to the rugged terrain and scarce resources within the study area. This research suggested that movement patterns and home range sizes might differ between male and female snow leopards, which may indicate different ecological needs and resource‐use techniques. Furthermore, this study provides reliable information on snow leopards from the telemetry data and links it to conservation implications in northeastern Nepal to ensure their long‐term survival, promote coexistence and foster cross‐border collaboration.

## Graphical abstract

We analysed spatiotemporal dynamics using satellite telemetry data to understand differences in movement patterns, activity budgeting and home range utilisation between male and female snow leopards, satellite‐collared in the northeastern Himalayas of Nepal. The movement behaviour and activity budgeting of snow leopards were modelled using a hidden Markov model (HMM), whereas home ranges were estimated and compared using multiple methods, such as the minimum convex polygon (MCP), the kernel density estimation method with href bandwidth (KDE href), and the Brownian bridge movement model (BBMM). This research suggested that movement patterns and home range sizes might differ between male and female snow leopards, which may indicate different ecological needs and resource‐use techniques, emphasising the significance of a landscape‐level approach to conservation.

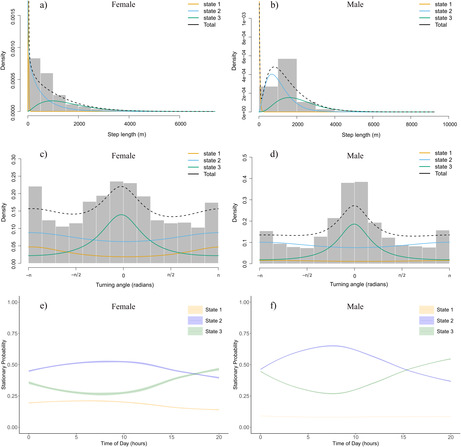

## 1 Introduction

The snow leopard ( *Panthera uncia* ), a crepuscular species inhabiting mountainous areas of Central and South Asia (Hussain 2003; McCarthy et al. 2008; Snow Leopard Network 2014) is an apex predator adapted as a solitary animal due to the sparse availability of its prey species in the challenging rugged terrain (Jackson 1996; Li et al. 2020). The large home range of snow leopards makes them a potential flagship and umbrella species of the mountain ecosystem of Asia (Snow Leopard Network 2014; Li et al. 2020). However, in recent years, the increasing threats to its survival due to habitat fragmentation, low prey density, poaching and climate change have been major reasons that have led to declining populations throughout its range (Hussain 2003; Wegge, Shrestha, and Flagstad 2012; Snow Leopard Network 2014; Chetri, Odden, and Wegge 2017; McCarthy et al. 2017; Li et al. 2020; Singh et al. 2022). Consequently, it is designated under the category of ‘Vulnerable’ in the IUCN red list (McCarthy et al. 2017). In Nepal, the snow leopard is listed as a protected mammal under the National Parks and Wildlife Conservation Act of 1973 and has been prioritised for protection.

Biologists face significant challenges when researching snow leopards because they do not occur in high densities (Sharma et al. 2021), their irregular movement patterns make them difficult to track (Yu et al. 2022) and they are extremely elusive with their coat pattern offering ideal camouflage, earning them the nickname the ghost of the mountains (Jackson 2020). Moreover, their preference for inhabiting cliffs and ridges in the rugged terrain, areas that improve their ability to spot prey or potential predators (Fox and Chundawat 2016), further complicates their study. While research on snow leopards in Nepal has mostly relied on people's perception (Hanson 2018; Shrestha et al. 2022; Shahi et al. 2023), diet and prey selection, and prey behaviours (Chetri, Odden, and Wegge 2017; Shrestha, Aihartza, and Kindlmann 2018), very few studies were carried out using VHF‐collars (Jackson and Ahlborn 1989; Jackson 1996; Oli 1997) and satellite collars (KCA 2019). While satellite telemetry study results have been reported from other snow leopard territories such as Pakistan (McCarthy et al. 2007), Mongolia (Johansson, Simms, and McCarthy 2016) and China (Yu et al. 2022), no comprehensive findings have been published except for a preliminary expedition report (KCA 2019), and movement ecology has been largely overlooked in the Himalayas of Nepal. Considering ongoing climate change and its threats to snow leopards and their habitat, understanding their movement behaviour becomes imperative (Berg et al. 2010; Beever et al. 2016; MoFSC 2017).

Tracking animal movements over time can reveal information about their behaviour, interactions and population dynamics (Hooten et al. 2017; Patterson et al. 2017) providing a better understanding of how animals forage, find mates, avoid predators, migrate and disperse on a range of spatial and temporal scales (Wang 2019). Animals don't merely move, but they also engage in other activities such as foraging or resting. The manner in which the animals move, such as how quickly they move, how far they move, and how tortuous their movement path is, can indicate what they might be doing (Shepard et al. 2008; Brown et al. 2013; Bennison et al. 2018). For example, slow movement with short step lengths and large turns may indicate resting or foraging, whereas fast movement with long step lengths and little turning may indicate travel (Morales et al. 2004). Sudden active movement followed by resting, with typical step lengths and turns, can indicate activities such as hunting. By examining step length and turning angle, we may be able to better comprehend these behavioural patterns (Wilson, Shepard, and Liebsch 2008; Nathan et al. 2012; Edelhoff, Signer, and Balkenhol 2016). Hidden Markov models (HMMs) are a popular method used to identify behavioural states in animal movement data such as in caribou (*Rangifer tarandus*) (Franke, Caelli, and Hudson 2004), minke whales (*Balaenoptera acutorostrata*) (Christiansen, Rasmussen, and Lusseau 2011), grey wolves (*Canis lupus*) (Ylitalo, Heikkinen, and Kojola 2021), Florida panthers (*Puma concolor coryi*) (Van de Kerk et al. 2015), lions (*Panthera leo*) (Goodall et al. 2019) and Persian leopards (*Panthera pardus saxicolor*) (Farhadinia et al. 2020). However, prior to this study, no such process‐based models had been used to investigate snow leopard movement behaviours. Home range mapping is another tool to expand knowledge regarding space use, behavioural activities and intra‐species interactions by estimating the area where an animal spends most of its time and core areas with concentrated use (Samuel, Pierce, and Garton 1985; Laver and Kelly 2008; Powell and Mitchell 2012). Previous home range estimations of snow leopards using GPS‐based surveys (Johansson, Rauset, et al. 2016; KCA 2019; Yu et al. 2022) were undertaken using approaches such as the minimum convex polygon (MCP), fixed kernel with a smoothing factor (KDE) and local convex hull (LOCoH), leaving gaps in understanding time‐dependant estimation of spatial use. Thus, in this study the Brownian bridge movement model (BBMM) was chosen for more accurate home range estimation along with two methods viz. MCP and KDE for comparison with prior studies in which BBMM was notably missing.

The analysis of the snow leopard's spatial ecology and behaviours forms the foundation for effective conservation, enabling the identification of crucial habitats, migration routes and breeding areas (Jackson and Ahlborn 1989; Fryxell, Sinclair, and Caughley 2014). Gaining real‐time insights into the movements and habitats of wildlife facilitate the formulation of targeted conservation plans, effective management of protected areas and mitigation of human–wildlife conflicts (Wegge, Shrestha, and Flagstad 2012) by identifying movement patterns and implementing measures to reduce interactions. Moreover, telemetry data promotes international collaboration, as it allows for information‐sharing across borders, leading to a broader understanding of species behaviours (Snow Leopard Network 2014; Fox and Chundawat 2016). Furthermore, it supports responsible tourism management, and educational outreach, and enhances the government's ability to make informed decisions for the coexistence of biodiversity and local communities (Wegge, Shrestha, and Flagstad 2012; Snow Leopard Network 2014; KCA 2019). To further strengthen conservation initiatives, this study leveraged the very first satellite‐collar spatial data from Nepal's eastern region. By analysing the movement behaviour and home range of snow leopards, we aimed to link these findings directly to conservation implications, providing reliable data to support sustainable management strategies for this iconic species.

## 2 Methods

### 2.1 Study Area

**FIGURE 1:**
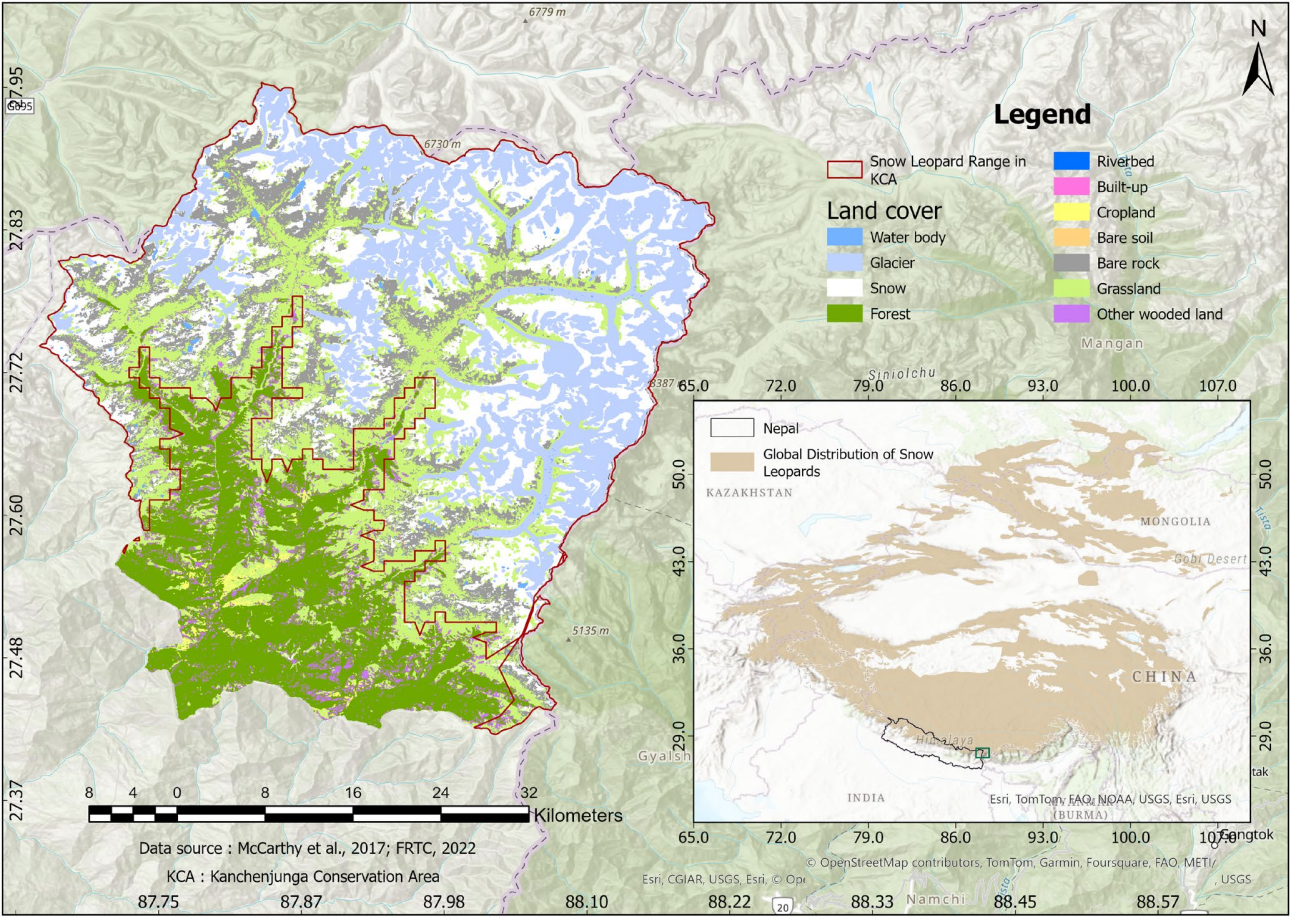
A map of the Kanchenjunga Conservation Area (KCA) in northeast Nepal, bordered by China to the north and India to the east, depicting land cover types, snow leopard distribution within the KCA and snow leopard distribution globally.

Kanchenjunga Conservation Area (KCA) is located in the northeastern part of Nepal in the Taplejung district (26°24′–27°56′ N and 87°39′–88°12′ E), covering an area of 2035 km^2^. It is named after Mount Kanchenjunga, Nepal's second‐highest peak and the world's third‐highest mountain at 8586 m asl. It borders Qomolangma National Nature Preserve in Tibet, China to the north and Khangchendzonga National Park in Sikkim, India to the east (Bhuju et al. 2007). The landscape is steep and rugged, with mountainous terrain (Figure 1). The climate in the area is alpine, sub‐alpine or mixed temperate depending upon the elevation range (below or over 3000 m asl). The major vegetations found in this area are Himalayan Larch (*Larix griffithiana*), Magnolia kisopa (*Michelia kisopa*), Nepali hog‐plum ( *Choerospondias axillaris* ) and *Aconitum spicatum*. The fauna such as snow leopard ( *Panthera uncia* ), leopard cat ( *Prionailurus bengalensis* ), brown bear ( *Ursus arctos* ), blue sheep ( *Pseudois nayaur* ), Tibetan wolf ( *Canis lupus* ) and Himalayan goral ( *Naemorhedus goral* ) are protected in this area (KCA 2023).

### 2.2 Snow Leopard Capturing and Handling (KCA 2019)

The satellite collaring of snow leopards was conducted by DNPWC (Department of National Parks and Wildlife Conservation, GoN, Nepal) in collaboration with WWF (World Wide Fund For Nature, Nepal), NTNC (National Trust for Nature Conservation, Nepal) and KCAMC (Kanchenjunga Conservation Area Management Council, Nepal) from 2013 to 2017 (KCA 2019). Firstly, the Snow Leopard Conservation Committee (SLCC) reconnaissance survey was done to locate appropriate capturing sites. In addition to that, sign surveys and camera traps were used to plot the snow leopard activity and potential trap layout strategies. Based on this information, traps were placed in strategic locations of snow leopard such as relic sites, kill sites, or frequently used trails. On average, 28 Aldrich‐style foot snare and/or Belisle foot snares connected with VHF or satellite transmitter were used in each expedition and were monitored remotely every 2 h to check whether traps were triggered.

When the snow leopard was caught in the trap, it was darted, from 7 to 15 m, on the rump by an Air pressure dart gun (Telinject Inc.) with a 3 mL syringe fitted with a 1.5 × 30 mm collared needle. A solution of Zoletil 100 (Tiletamine 250 g + Zolazepam 250 g) and medetomidine (Sedator 1 mg/mL) [5 mL of Sedator to one vial of Zoletil] was administered, based on their body weight for both adults and sub‐adults, for 45–60 min anaesthesia. Subsequently, the morphological and physiological data were recorded and were used to determine the individual's approximate age. The animal was then fitted with satellite GPS collars based on either iridium or Globalstar communication platform. The automatic and radio‐controlled drop‐offs were set to the collar. It was set to store 4‐h location data (GPS fix rate) on board and to send to a workstation in Kathmandu via networks of satellites. Finally, to revive the snow leopard, atipamezole hydrochloride (antisedan 5 mg/mL) was injected intramuscularly at a dose five times higher than medetomidine (the detailed process is provided in KCA 2019).

### 2.3 Data Analysis

The telemetry data of five satellite‐collared snow leopards were retrieved from the DNPWC in 2024, out of which the data of one snow leopard was discarded because the signal was interrupted after 13 days, and the collar failed after 21 days resulting in a total of 72 location points, which were insufficient for analysis. The Elevation data were obtained by the collar, whereas the other covariates such as Slope and Aspect were retrieved from the Digital Elevation Model (DEM) raster data with a spatial resolution of 30 m for that area using the Shuttle Radar Topography Mission (SRTM) website (https://srtm.csi.cgiar.org/srtmdata/) and ArcGIS v.10.8 (ESRI 2021).

For studying the movement ecology of wildlife, the hidden Markov model (HMM) is one of the widely utilised state‐space models due to its adaptability and efficient statistical analysis techniques, which are built on the concept of a discrete latent process (Hooten et al. 2017). It focuses on a theory where a chain of actions occurs, with each action dependent on the one immediately preceding it and not on any earlier actions (Hooten et al. 2017). HMM is a time series model consisting of two parts: an observable time series, that is, the animal's actual movement, and a hidden state sequence (State 1, State 2, State 3, etc.), that is, the animal's movement behaviour and time spent on each of the states which cannot be directly perceived. Those state sequences of animals are related to their behaviour such as resting, foraging, hunting, travelling, etc. Each behaviour is characterised by step length (distance between two consecutive relocations; m) and turning angle (angle between the current displacement and the previous displacement; radian), while activity budgeting (proportion of time spent in each behavioural state) can be derived from the fitted HMM.

In this study, the movement behaviour of male and female snow leopards was modelled separately using HMMs for 2‐state and 3‐state models, both with and without covariates such as elevation, slope, aspect, time of day and their combinations. These covariates have an effect on the probability that the animal is in certain behavioural states. The time of day was not normalised, given that the variation in sunrise and sunset times in the area is minimal. In the 2‐state movement model, State 1 was characterised by undirected, shorter step lengths (resting phase) and State 2 was characterised by directed, longer step lengths (travelling phase). The 3‐state movement model anticipated three distinct movement patterns. State 1 was expected to have short step lengths and high turning angles (close to −π or π), indicating slow, undirected movement during a resting phase. State 2 was expected to have moderate step lengths and relatively low turning angles, indicating moderately active movement. Finally, State 3 was expected to have long step lengths and small turning angles (near zero), indicating fast, directed movement associated with a travelling phase.

Hidden Markov models require discrete‐time data for fitting. These models are fitted using numerical maximisation of the likelihood function, which requires an initial set of parameter values to begin the iterative optimisation process (Michelot and Langrock 2023). The initial parameters were defined after visualising the data: observed mean and standard deviation for step lengths and the observed mean and inverse of the variance for turning angles on a histogram for each state in both 2‐state and 3‐state behaviours (Michelot and Langrock 2023). These parameters enable the evaluation of the likelihood of the observed data, revealing all possible state sequences that could adequately describe the observed time series (Pohle et al. 2017; Patterson et al. 2019). However, in actuality, data frequently arrives at irregular intervals, providing problems for HMM operation. To address this issue in this study, missing relocation data (36.67% of total data) were imputed at four‐hour time intervals using the *crawl* package (Johnson and London 2018; Togunov et al. 2021). Following that, the *momentuHMM* package (Mcclintock and Michelot 2018) was used to model the behavioural states. AIC and BIC values for all the models were compared to find the best‐fitted model. The pseudo‐residual plots were plotted to check for the goodness of fit of the model and stationary plots were plotted to graphically represent their stationary probabilities in each of the states.

Home range calculation measures not only include positional data (as in minimum convex polygon) but also account for its space use density in normally distributed bivariate space (as in kernel density estimation) and, is extended to use a conditional ‘random walk’ process while moving from one location to another (as in Brownian bridges), resulting in more accurate quantification of home ranges (Bevanda et al. 2016). Multiple methods were used to estimate the home range of snow leopards. For this, the location data containing a DOP (dilution of precision) greater than 5.2 were filtered out, as data with higher DOP values indicate reduced location accuracy (Ironside et al. 2017). This filtering process resulted in the retention of 82.65% of the original dataset, ensuring that only the more reliable location data were used in the home range analysis. For every individual, the minimum convex polygon (MCP), kernel density estimation (KDE href) method with smoothing parameter; href bandwidth (Ghangjenjwenga = 2715.57 m, Lapchhemba = 4759.66 m and Yalung = 2894.3 m) and Brownian bridge movement model (BBMM) were used to analyse their home range at the 95% and 50% isopleths using the *adehabitatHR* package (Calenge 2011) in R platform v. 4.3.2 (R Core Team 2023). BBMM technique relies on two crucial parameters: the uncertainty of the assumed locations (sig2) and a smoothing factor associated with the animal's movement speed (sig1). In this case, we chose 30 m of sig2 and the calculated sig1 using the *liker* function from the *adehabitatHR* package was 7.9. To represent the spatial extent of home ranges and overlap between male and female snow leopards, vector data (polygon shapefiles) were generated for each method and then plotted on an NDVI map of the same period obtained from the Google Earth Engine dataset MOD13Q1.061 (https://code.earthengine.google.com/) in ArcGIS v.10.8 (ESRI 2021). However, the overlaps may not represent the same shared space at the same time, as snow leopards were not collared simultaneously.

## 3 Results

A total of five successful satellite collars were placed on snow leopards in Kanchenjunga from 2013 to 2017. In the first two expeditions in 2013 and 2014, the same adult male, Ghangjenjwenga, was captured twice. Thus, in the second capture, the first collar was successfully retrieved, and a second collar was installed (KCA 2019). Though it had two IDs, the location data were pooled for this study since it was from the same individual. Omi Khangri, another adult male, was captured and collared in 2015, however, the signal was lost and then found again multiple times before it was finally lost (KCA 2019). So, the location data collected for it was considered insufficient and discarded from the further analysis in this study. Lapchhemba and Yalung, both sub‐adult females, were captured and collared in 2016 and 2017, respectively, with only 16 days of temporal overlap between them. Hence, this study used only three individuals (one male and two female snow leopards) for the telemetry analysis. The snow leopards were observed across a wide elevation range, from a minimum of 2040.22 m asl to a maximum of 5858.81 m asl. The capture effort (number of days to capture the particular snow leopard) varied from 30 h to 33 days. These individuals were captured and collared from mid‐spring to late autumn. Among the three individuals, the male snow leopard was observed for 659 days, resulting in 1871 GPS fixes, whereas two female snow leopards were monitored for 392 and 168 days, respectively, yielding 1983 and 781 GPS fixes recorded at four‐hour intervals. The brief descriptions of each captured snow leopard are summarised in the table (Table 1).

**TABLE 1:** A brief description of satellite collared snow leopards in Kanchenjunga Conservation Area, Nepal.

Name/ID	Sex/age	Weight (kg)	Capture date	Last fix date	Number of days	Number of location points
Ghangjenjwenga/13646	M/5	40	2013‐11‐26	2014‐05‐15	171	476
Ghangjenjwenga/13647	M/~6	40	2014‐05‐16	2015‐09‐15	488	1395
Omi Khangri/17103^a^	M/4–5	41	2015‐05‐21	2015‐06‐10	21	72
Lapchhemba/21245	F/2–3	30	2016‐04‐28	2017‐05‐24	392	1983
Yalung/21755	F/2	30	2017‐05‐08	2017‐10‐22	168	781

^a^ Data not used in analysis.

### 3.1 Movement Behaviour

Among all the HMMs for both male and female snow leopards, the top model based on both AIC and BIC was the model with single covariate, time of day (tod), demonstrating that time of day was an essential variable in identifying the three behavioural states of snow leopards. In contrast, biophysical terrain factors such as slope, aspect and elevation had no effect on discriminating between the three states (Table 2).

**TABLE 2:** Lower AIC and BIC values imply better model fit. The hidden Markov models (HMMs) were fitted separately for male and female snow leopards, allowing for comparisons of sex‐specific behaviours. Each HMM integrated different covariates into the hidden process to determine their impact on movement patterns.

Models	AIC		ΔAIC		BIC		ΔBIC	
	Female	Male	Female	Male	Female	Male	Female	Male
3 state + tod	58,237.36	65,462.73	0	0	40,904.17	65,629.19	0	0
3 state + slope	58,390.97	65,474.56	153.61	11.83	41,033.43	65,968.74	129.26	339.55
3 state + tod + slope	58,399.72	65,482.39	162.36	19.66	41,112.63	65,683.42	208.46	54.23
3 state	58,481.54	65,884.64	244.18	421.91	42,996.51	77,534.92	2092.34	11,905.73
3 state + aspect	58,809.23	65,805.4	571.87	342.67	58,676.23	65,637.9	17,772.06	8.71
3 state + slope + aspect	58,818.92	65,476.89	581.56	14.16	41,154.1	65,677.92	249.93	48.73
2 state	58,886.78	66,421.14	649.42	958.41	43,183.5	78,056.31	2279.33	12,427.12
3 state + elevation	59,599.26	66,113.37	1361.9	650.64	41,305.93	66,282.75	401.76	653.56
3 state + tod + slope + elevation	59,869.16	66,226.7	1631.8	763.97	41,194.65	66,465.43	290.48	836.24
3 state + elevation + slope	61,710.19	66,222.79	3472.83	760.06	41,146.54	66,423.82	242.37	794.63

Abbreviation: tod, time of day.

**FIGURE 2:**
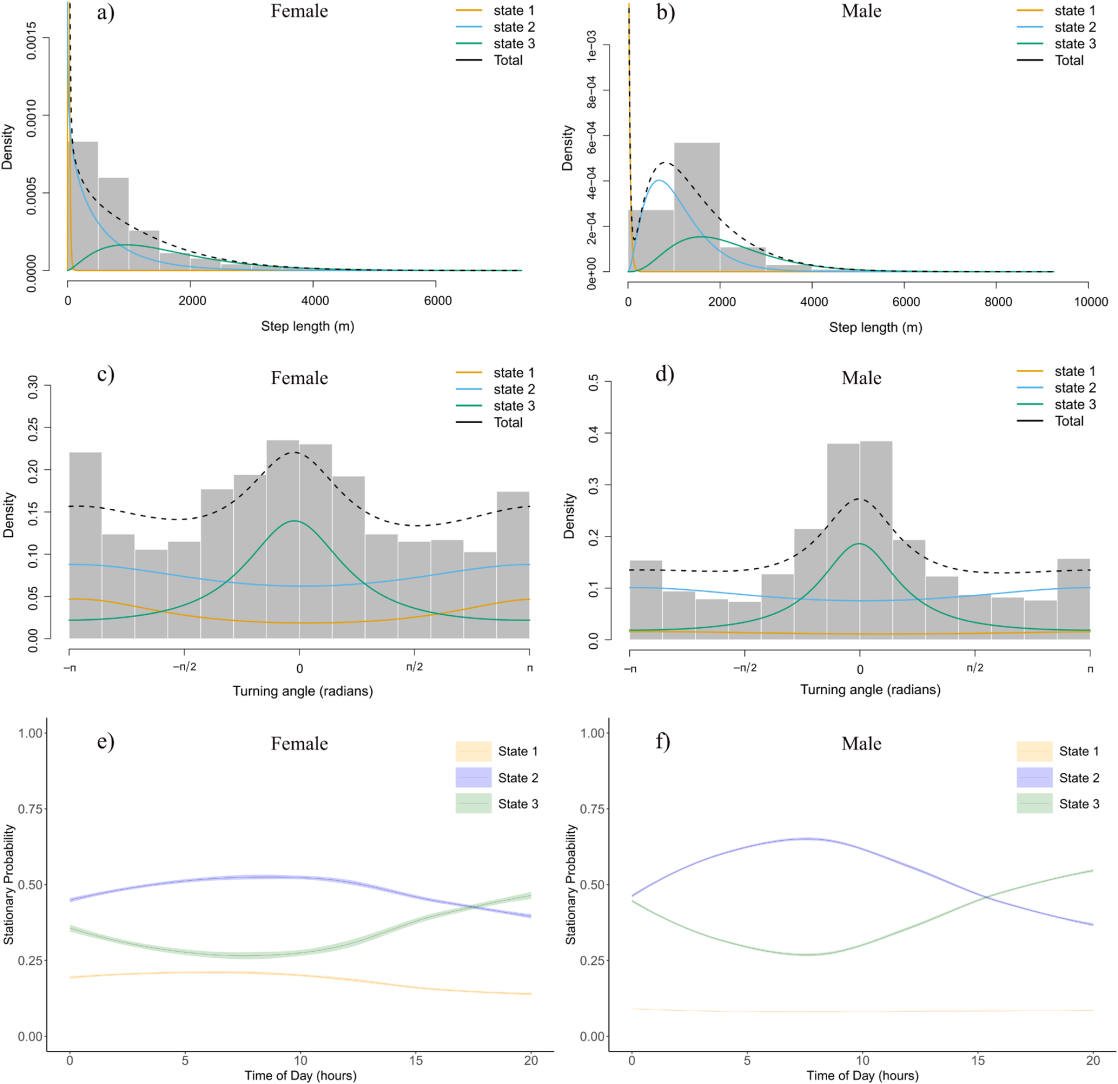
State‐dependent distributions for step lengths (a, b), turning angles (c, d) and stationary state probabilities as a function of time of day (e, f), with 95% confidence intervals, estimated for male (*n* = 1) and female (*n* = 2) snow leopards across three behavioural states: State 1 (resting), State 2 (moderately active) and State 3 (travelling).

The 3‐state movement behaviour estimation of three individual snow leopards demonstrated that their movement patterns varied across each state. In each behavioural state, the mean step length of the male was greater than that of females. In State 1, the mean step length for females was 13.97 m (±12.13) with mean turning angle of −2.945 (concentration parameter *k* = 0.221) whereas for the male, the mean step length was 29.31 m (±30.97) with mean turning angle of −2.785 (concentration parameter *k* = 0.079). Similarly, in State 3, the mean step length was 1501.68 m (±1005.25) with mean turning angle of −0.082 (concentration parameter *k* = 0.377) for females and 2150.1 m (±1062.16) with mean turning angle of −0.013 (concentration parameter *k* = 0.513) for the male. The highest absolute turning angle combined with the lowest concentration in State 2 indicated that it was the most undirected movement for both male and female snow leopards. In contrast, State 3 was the most directed movement, as evidenced by the lowest absolute turning angle and most concentration during this behaviour (Table 3 and Figure 2).

**TABLE 3:** Estimates of movement metrics and temporal activity budget for male and female snow leopards based on the best‐fitted model in each state: State 1 (resting), State 2 (moderately active) and State 3 (travelling).

	State 1	State 2	State 3
*Female*			
Step length mean (m)	13.97	543.95	1501.68
Step length sd (m)	12.13	655.43	1005.25
Angle mean (radians)	−2.945	3.072	−0.082
Angle concentration (*k*)	0.221	0.166	0.377
Activity budget	0.156 (0.144–0.171 ± 0.006)	0.394 (0.345–0.445 ± 0.023)	0.449 (0.396–0.503 ± 0.025)
*Male*			
Step length mean (m)	29.31	1062.08	2150.1
Step length sd (m)	30.97	654.14	1062.16
Angle mean (radians)	−2.785	−3.103	−0.013
Angle concentration (*k*)	0.079	0.068	0.513
Activity budget	0.086 (0.080–0.092 ± 0.002)	0.526 (0.443–0.609 ± 0.039)	0.387 (0.303–0.478 ± 0.041)

Moreover, female snow leopards allocated 15.6% of their activity budget in resting and 39.4% in moderately active state, while most of their activity budget, at 44.9%, was spent in travelling. In the case of the male, the activity budget revealed that the greatest proportion of time was allocated to moderately active state (52.6%), followed by travelling (38.7%) (Table 3).

Both male and female snow leopards showed periodic activity based on the time of day. The probability of being in a travelling state was lower at dawn than at dusk. In contrast, the probability of being moderately active was greater at dawn and decreased during the day. Resting state was nearly unaffected by time of day (Figure 2).

The *Q*–*Q* plot of both the step length and turning angle pseudo‐residuals showed that the residuals followed a normal distribution, providing evidence to support the model's goodness of fit. Additionally, the autocorrelation function showed a few statistically significant autocorrelations, suggesting that the movements of male and female snow leopards were unlikely to have occurred randomly (Figure S1).

### 3.2 Home Range

The home range of all satellite‐collared snow leopards were widely dispersed across the landscape, crossing the international boundaries, suggesting that each individual occupies an extensive and scattered home range.

**FIGURE 3:**
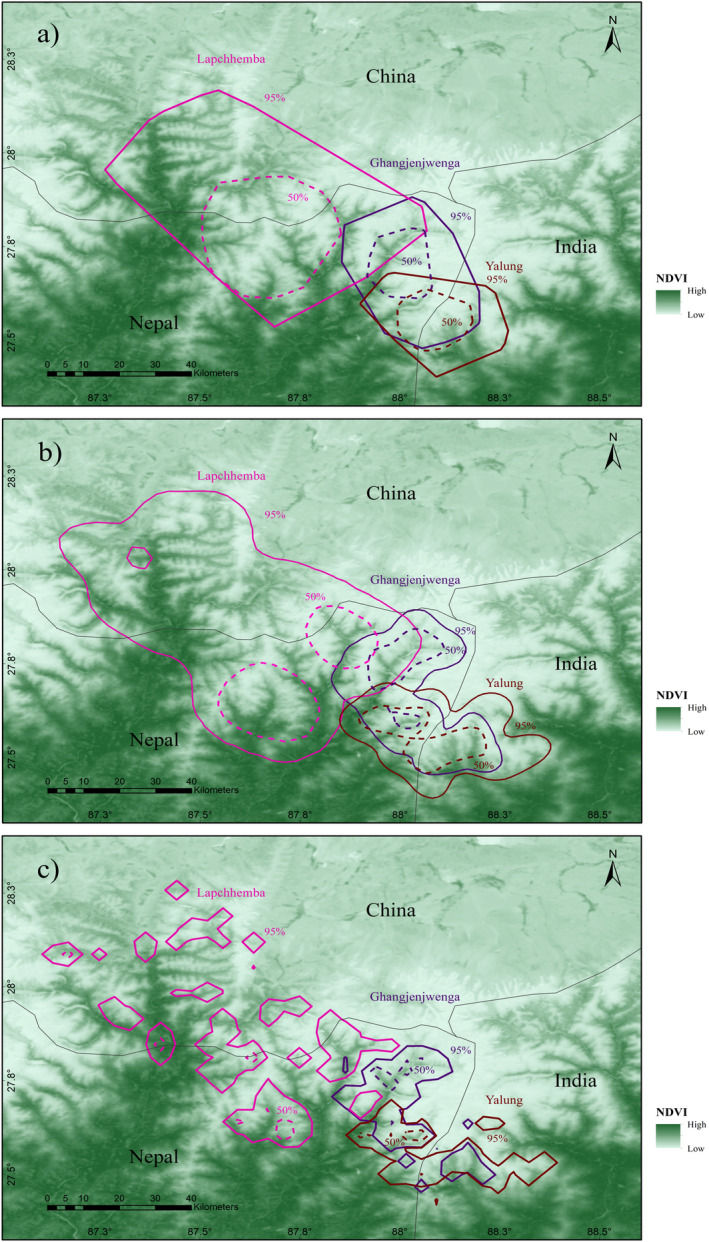
Spatially explicit home range map of three snow leopards, based on individual tracking periods: 659 days for Ghangjenjwenga (adult male, ID 13646 and 13647), 392 days for Lapchhemba (subadult female, ID 21245) and 168 days for Yalung (subadult female, ID 21755). Home ranges are depicted at 95% and 50% isopleths and were estimated using: (a) Minimum Convex Polygon (MCP), (b) Kernel Density Estimation with href bandwidth (KDE href) and (c) Brownian Bridge Movement Model (BBMM). The map highlights international boundary crossings from Nepal into India and China, as well as the extent of range overlap among the individual snow leopards. However, the individuals may not be sharing the same space at the same time, as they were not collared simultaneously.

In the MCP, the home range varied from 726.20 to 3005.16 km^2^ whereas, in KDE href from 1033.97 to 3775.33 km^2^ and in BBMM from 432.49 to 1023.56 km^2^. The KDE href produced the largest estimates for all the individuals, particularly for Lapchhemba whose 95% KDE href was 3.69 times larger than the equivalent BBMM area (Table 4). The 50% isopleth area for each method represents the core area within the home range, which was two to nine times smaller than the corresponding 95% areas for the respective individuals. Among three individuals, Lapchhemba had the wider home range followed by Ghangjenjwenga with some overlapping home range with one another (Figure 3). The area overlaps between Ghangjenjwenga and Yalung exceeded 20% in the 95% isopleths across all home‐range estimation methods, while their overlap was limited to 5% in the 50% isopleths. In contrast, Yalung and Lapchhemba had minimal overlap, with only 0.24% in the 95% isopleth using the KDE href method, and no overlap in any of the core areas. A spatially explicit map of home ranges in Figure 3 showed that the collared snow leopards had home ranges that extended beyond Nepal's borders. Lapchhemba's home range and core area stretched across Nepal and China. Similarly, Ghangjenjwenga and Yalung's ranges extended across Nepal and India. Yalung's core area was shared by Nepal and India, whereas Ghangjenjwenga's core area was entirely in Nepal.

**TABLE 4:** Home range (in km^2^; based on individual tracking period; 659 days for Ghangjenjwenga, 392 days for Lapchhemba and 168 days for Yalung) of satellite‐collared snow leopards, using Minimum Convex Polygon (MCP), Kernel Density Estimation with href bandwidth (KDE href) and Brownian Bridge Movement Model (BBMM) at 95% and 50% isopleths in Kanchenjunga Conservation Area (KCA), Nepal.

Name/ID	MCP		KDE href		BBMM	
	95%	50%	95%	50%	95%	50%
Ghangjenjwenga/13646 and 13647	1048.43	263.35	1073.09	217.31	518.99	72.08
Lapchhemba/21245	3005.16	954.75	3775.33	694.12	1023.56	115.33
Yalung/21755	726.20	253.88	1033.97	291.88	432.49	57.67
*Area overlap*						
Ghangjenjwenga and Lapchhemba	303.17	0.00	313.12	1.61	39.74	0.00
Ghangjenjwenga and Yalung	465.00	7.49	577.13	24.33	215.28	0.00
Lapchhemba and Yalung	0.00	0.00	11.52	0.00	0.00	0.00

*Note:* The individuals were not collared simultaneously, so they may not be sharing the same space at the same time.

## 4 Discussion

### 4.1 Movement Behaviour

This study is the first to use GPS‐based satellite telemetry data to analyse the movement behaviour of snow leopards in the eastern region of Nepal. The agreement between AIC and BIC provided strong evidence that snow leopard movement is best explained by three behavioural states: resting, moderately active and travelling. However, it should be noted that there are likely many more nuanced behaviours not captured in this model. Jackson (1996) investigated snow leopard movement using VHF transmitters, assessing linear distance measures and activity patterns based on pulse rate variations and changes in signal strength. While VHF collar data can determine whether a snow leopard is resting or moving, it cannot reveal specific behavioural states such as moderately active or travelling. GPS‐based satellite collar data analysed with a hidden Markov model (HMM) was able to observe such behavioural states. Step length and turning angle, derived from movement data, are incorporated into the models. When combined, these metrics help identify key behavioural stages during animal movements, rather than simply distinguishing between generic activity patterns such as active or inactive states.

The step lengths and the turning angle of both male and female differed. The male had longer step lengths and lower turning angles in each movement behaviour state compared to females, suggesting that the two sexes employed different strategies for resource utilisation. While resting or foraging behaviours usually involve the most undirected movement, as seen in grey wolves (Ylitalo et al. 2021), this study discovered an unexpected pattern in snow leopards. Both male and female snow leopards showed the most undirected movement in moderately active state, which is similar to the behaviour of Persian leopard roaming around Iranian residents (Farhadinia et al. 2020). Furthermore, the model suggested that three distinct states in snow leopard were affected by the time of day, but not by slope, aspect and elevation. Similar results were extracted by Jackson (1996) regarding the relationship of activity pattern of snow leopards with time of day in central Himalayas of Nepal. However, factors like slope, aspect, elevation and ruggedness significantly influence home range and habitat selection strategies (Fox and Chundawat 2016; Jianhui et al. 2023; Rosenbaum et al. 2023).

Among the three‐movement states, the probability of travelling a longer distance, that is, State 3 was higher during dusk (18:00 h) and continued into the following night, while moderate movements, that is, State 2 were more frequent at dawn (06:00 h) with activity continuing for several hours during midday, as supported by findings of several studies (Jackson 1996; Oli 1997; McCarthy, Fuller, and Munkhtsog 2005; McCarthy et al. 2017; Johansson et al. 2022). The proportion of time spent in the resting phase, that is, State 1 was less for the male than for females. Moreover, the male spent more than half the time in moderately active movement, that is, State 2 in its permanent home range while females spent the majority of their time in highly active movement, that is, State 3. The longer period of moderate activity may reflect their behaviours such as hunting, frequent visits to kill sites, patrolling and searching for mates during the breeding season (Grönberg 2011; Fox and Chundawat 2016; Krofel et al. 2021). In contrast, the exploratory behaviour of females may be attributed to their younger age, as they may still be refining their home range while developing hunting skills (Jackson 1996; Snow Leopard Network 2014; Fox and Chundawat 2016; Johansson et al. 2018, 2022). Despite a general grasp of snow leopards' behavioural ecology, there was little information about their behaviour in Nepal until this study. This disparity makes it difficult to understand their reactions to landscape and climate change. As a result, monitoring their travel patterns, ecology, behavioural phases and activity budgeting at regular intervals can greatly assist protected area managers with developing measures to offset the consequences of climate change and other disturbances.

### 4.2 Home Ranges Estimates

This study indicated that snow leopards' estimated average home ranges were substantially larger than those previously reported utilising VHF radio collars in Nepal (Jackson and Ahlborn 1989; Jackson 1996; Oli 1997) and GPS‐based collar in other countries (Johansson, Rauset, et al. 2016; Yu et al. 2022; Rosenbaum et al. 2023). Five radio‐collared snow leopards in ideal habitat in the Nepalese Himalaya had home ranges of 11–37 km^2^ (Jackson 1996). Additionally, research conducted on satellite‐tracked snow leopards in China discovered that GPS‐collar equipped adult male had an average home range of 47–838 km^2^, whereas adult females' occupied regions ranged from 174 to 872 km^2^ using 95% MCP, 95% KDE and LoCoH methods (Yu et al. 2022).

The home range estimate of BBMM for the male snow leopard of Nepal, that is, Ghangjenjwenga was 2.36 times larger than the mean home range size based on the LoCoH estimates for adult male snow leopards in Mongolia, and that for Yalung, a sub‐adult female snow leopard of Nepal was 2.05 times larger than the mean for sub‐adult female snow leopards in Mongolia (Johansson, Rauset, et al. 2016). However, the large home range of Lapchhemba was due to its exploring age. Since it had recently separated from its mother (KCA 2019), it went on its own to explore and navigate the areas to make its home range (Jackson 1996; Fox and Chundawat 2016; Johansson et al. 2018). Ghangjenjwenga, being an adult, had already explored and had its pre‐designated home range, thus, its home range was smaller as compared to Lapchhemba. Also, one of the determinants of the home range of an individual animal is the availability of resources. The animal ranges less in the area with sufficient resources and little conspecific competition (Shrestha, Aihartza, and Kindlmann 2018; Oberosler et al. 2022; Sharief et al. 2022; Rosenbaum et al. 2023). In areas with limited prey availability, snow leopards may need wider hunting territory. This could explain snow leopards' larger home ranges in the eastern Himalayas, where prey is scarcer. For instance, the Kanchenjunga Conservation Area had only 2000 blue sheep in 2016 across 2035 km^2^, which is a small number when compared to Shey Phoksundo National Park, Nepal, which had 6167 blue sheep in 2022 across 3555 km^2^ (Shrestha 2017, March 17, SPNP 2023).

The home range of the male snow leopard had a large area of overlap with those of females whereas the females had negligible overlap with each other. The previous studies (Jackson and Ahlborn 1989; Jackson 1996; Oli 1997; Johansson, Simms, and McCarthy 2016; Johansson et al. 2018; Rosenbaum et al. 2023) also showed some pattern of home range overlap of snow leopards. In the study conducted by Johansson, Rauset, et al. (2016), male–female home range overlap was less than 20%, as was the case between Ghangjenjwenga and Yalung, however, it was more than 20% between Ghangjenjwenga and Lapchhemba. The range of overlapping depends on the age, sex, reproduction season and kinship (Snow Leopard Network 2014). Males tend to overlap more female home ranges to increase access to mating (Goodrich et al. 2010; Johansson et al. 2018). In the case of females, the subadults tend to overlap their mother's home range (Johansson et al. 2021, 2024), while adults prioritise resources for themselves and their cubs, often overlapping with more than one male to choose the better mate (Johansson et al. 2018).

Johansson, Rauset, et al. (2016) observed that about 40% of the 170 protected areas in snow leopard range countries are smaller than the home range of a single adult male snow leopard. Considering the larger home ranges reported in the current study, this percentage would likely increase further, emphasising the need for more extensive conservation areas. This research provides vital information to inform the redesign of smaller protected areas, such as expanding their size, creating suitable wildlife corridors or closely monitoring snow leopard movement patterns to protect them from threats like poaching. Moreover, it highlights the importance of adaptive management to ensure the long‐term survival of snow leopards in increasingly fragmented landscapes.

Admittedly, this study only considered one adult male individual for the behaviour and home range comparison with the younger females. So, the comparison was both gender and age based. The home range of sub‐adult females may not accurately represent that of adult females, as reproductive and parental care phases significantly influence home range utilisation in the latter (Johansson et al. 2018, 2022; Pålsson 2022). Therefore, further study with more individuals of different sexes and age groups is needed so that a clearer comparison could be done. Similarly, it is recommended to investigate how snow leopards use space within their home range by incorporating additional contextual data such as land cover, environmental data, prey distribution and other relevant variables.

### 4.3 Transboundary Cooperation

Nepal shares international boundaries with China and India, and snow leopard habitats observed in Nepal frequently overlap one or both bordering nations. In this study, the collared individuals crossed the international borders at northern and eastern sides of Nepal and went extensively to China, and India, underlining the importance of landscape‐level planning and cross‐border collaboration in conservation. Previous research, such as Forrest et al. (2012), had advised using landscape conservation planning techniques for snow leopards due to their large home ranges and the importance of overall biodiversity conservation in the region. The much broader home ranges validated the snow leopard's wide‐ranging behaviour. As a result, their conservation would be possible from a landscape‐level approach in which protection efforts should be integrated into multi‐use landscapes.

Nepal and China signed a Memorandum of Understanding (MoU) in 2010 to cooperate in the conservation of the region's biodiversity, which includes provisions for frequent meetings between high‐ranking officials from both nations (DNPWC 2017). Furthermore, authorities from protected areas (PAs) and district forest offices near the border periodically meet with their counterparts to discuss field‐level issues. However, such cooperation with India has not been established through official channels yet. In the future, it is necessary to develop an institutional arrangement to strengthen this transboundary cooperation. However, in this regard, the Global Snow Leopard and Ecosystem Protection Program (GSLEP) is a significant initiative to protect snow leopard populations across large landscapes. By focusing on community‐based conservation and sustainable development, GSLEP takes a holistic approach to safeguard this iconic species and its associated ecosystems (Snow Leopard Working Secretariat 2013). This study provided important information for organisations such as GSLEP, and other concerned authorities to shape their conservation actions in this study area. Overall, specific knowledge of movement, and the home range is needed to inform the layout of management and conservation interventions, particularly to assess the gap in protection plans, make species‐specific conservation strategies, re‐design protected areas and to maintain the cross‐border cooperation to secure long‐term survival and coexistence (Jackson 1996; Ale, Shah, and Jackson 2016; DNPWC 2017; MoFSC 2017; Li et al. 2020).

## 5 Conclusion

To enhance our knowledge of snow leopard movement patterns and home range dynamics, larger sample sizes are necessary for comparisons between males and females. However, this study shed light on the movement behaviours and activity budgeting of male and female snow leopards within the Kanchenjunga Conservation Area (KCA), Nepal. The findings indicated that the snow leopards in the KCA exhibit exploratory behaviour, particularly juveniles, resulting in significantly larger home ranges compared to previous estimates from other regions. This exploratory behaviour highlights the need for extensive or connected protected areas to accommodate their spatial requirements. The explicit maps of home ranges revealed that the ecological territories of this iconic species extend beyond the international borders. Therefore, we recommend that all nations with snow leopard habitats focus on protecting transboundary habitat corridors through joint conservation efforts. Thus, these findings have substantial implications for landscape‐scale conservation. Considering future threats, this new information about this vulnerable species, inhabiting one of the most difficult terrains, is critical for its conservation as an apex predator that contributes to regional biodiversity and ecological balance.

The extensive location data collected via GPS collars are suitable for individual‐level inquiries; however, to address population‐level questions about general space use, satellite collar data from multiple animals is needed. This study indicates that combining GPS‐based high‐resolution biotelemetry data with contemporary models such as hidden Markov models (HMMs) and Brownian bridge movement models (BBMMs) can provide accurate and novel insights. It is essential to prioritise conservation planning and efforts based on accurate and up‐to‐date species movement patterns and home range estimates.

## Author Contributions

**Pratistha Shrestha:** conceptualization (equal), formal analysis (equal), software (equal), validation (equal), writing – original draft (lead), writing – review and editing (equal). **Dayaram Pandey:** conceptualization (supporting), data curation (equal), validation (equal), writing – review and editing (equal). **Pemba Sherpa:** conceptualization (supporting), data curation (equal), validation (equal), writing – review and editing (equal). **Prakash Shah:** conceptualization (supporting), data curation (equal), validation (equal), writing – review and editing (equal). **Dipesh Kumar Sharma:** conceptualization (equal), formal analysis (equal), software (equal), validation (equal), writing – original draft (supporting), writing – review and editing (equal).

## Conflicts of Interest

The authors declare no conflicts of interest.

## Supporting information


Figure S1

## Data Availability

Data are available at the Github Repository: https://github.com/DipeshDFRS/Snow_leopard.
