# Supplementary material for: Tracking the Ghosts of the Himalayas: Snow Leopard Conservation Insights From Satellite Collar Data
Source: Ecol Evol. 2025 Jan 6;15(1):e70802. doi: 10.1002/ece3.70802 (PMC11705456; doi:10.1002/ece3.70802)
Supplement: Supplementary file 1 — Figure S1 [file ECE3-15-e70802-s001.docx]

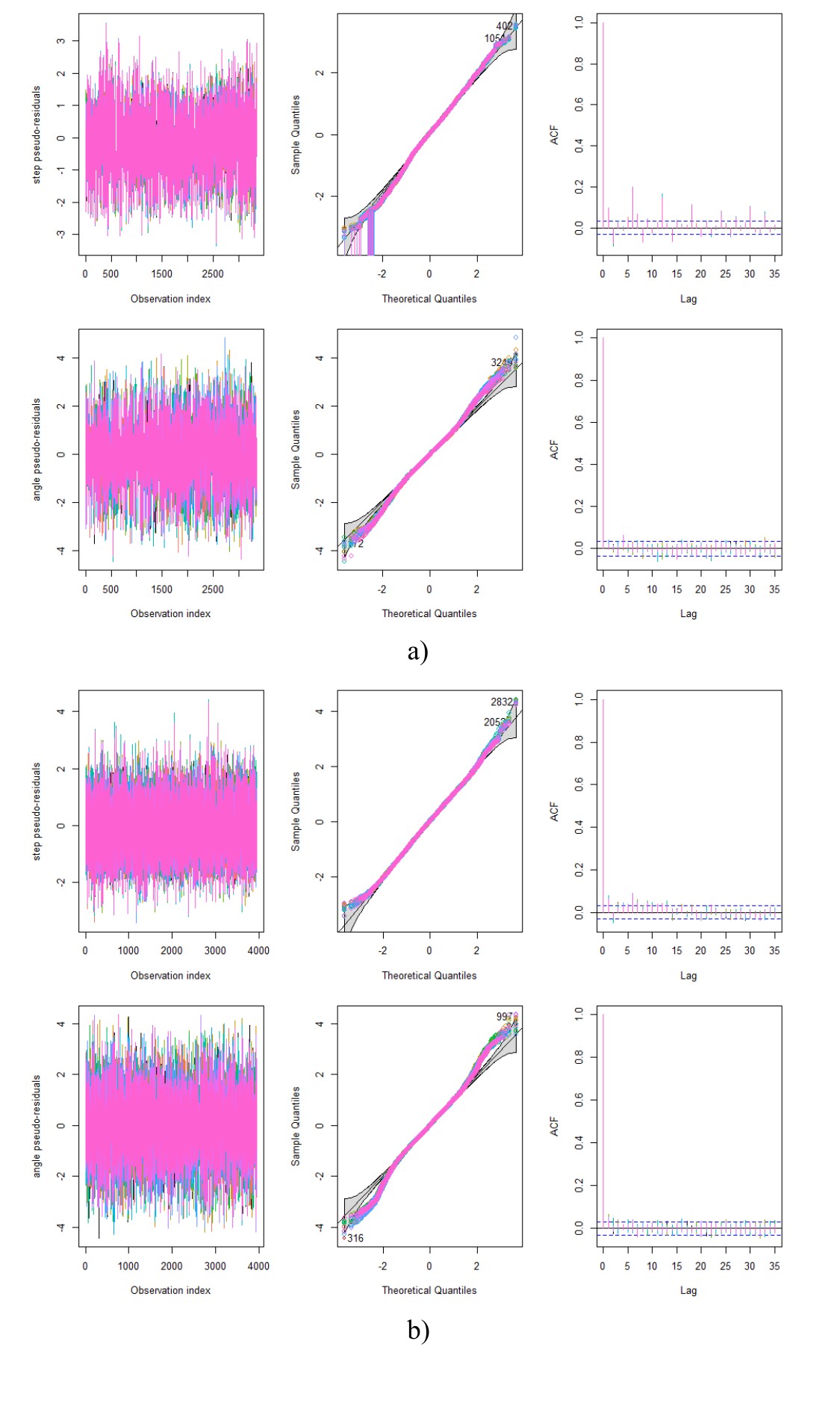


Figure S1: Quantile-quantile plots and autocorrelation functions of normal pseudo-residuals for step lengths and turning angles of a) female; b) male snow leopards, with different colours representing the results from each imputed model.
